# Microneedle pretreatment enhances the percutaneous permeation of hydrophilic compounds with high melting points

**DOI:** 10.1186/2050-6511-13-5

**Published:** 2012-08-13

**Authors:** Jessica Stahl, Mareike Wohlert, Manfred Kietzmann

**Affiliations:** 1 Department of Pharmacology, Toxicology and Pharmacy, University of Veterinary Medicine Hannover, Foundation, Buenteweg 17, Hannover, 30559, Germany

**Keywords:** Transdermal drug delivery, Microneedles, logK_ow_, Melting point, Non-steroidal anti-inflammatory drug, *In vitro* permeation study, Physical penetration enhancement

## Abstract

**Background:**

Two commercially available microneedle rollers with a needle length of 200 μm and 300 μm were selected to examine the influence of microneedle pretreatment on the percutaneous permeation of four non-steroidal anti-inflammatory drugs (diclofenac, ibuprofen, ketoprofen, paracetamol) with different physicochemical drug characteristics in Franz-type diffusion cells. Samples of the receptor fluids were taken at predefined times over 6 hours and were analysed by UV–VIS high-performance liquid-chromatography. Histological examinations after methylene blue application were additionally performed to gather information about barrier disruption.

**Results:**

Despite no visible pores in the *stratum corneum*, the microneedle pretreatment resulted in a twofold (200 μm) and threefold higher (300 μm) flux through the pretreated skin samples compared to untreated skin samples for ibuprofen and ketoprofen (LogK_ow_ > 3, melting point < 100°C). The flux of the hydrophilic compounds diclofenac and paracetamol (logK_ow_ < 1, melting point > 100°C) increased their amount by four (200 μm) to eight (300 μm), respectively.

**Conclusion:**

Commercially available microneedle rollers with 200–300 μm long needles enhance the drug delivery of topically applied non-steroidal anti-inflammatory drugs and represent a valuable tool for percutaneous permeation enhancement particularly for substances with poor permeability due to a hydrophilic nature and high melting points.

## Background

The topical transdermal administration of systemically active drugs represents a convenient alternative to systemic administration via oral route in both humans and animals with many advantages like the avoidance of the first-pass hepatic metabolism, enzymatic degradation and side effects in the gastro-intestinal tract. The outmost layer of the epidermis, the *stratum corneum*, plays a key role in the skin barrier concerning the intrusion of foreign substances from the environment and transepidermal water loss (TEWL) [1]. It is composed of keratin containing corneocytes embedded in a lipid rich matrix, which acts like a kit-substance and mainly comprises ceramides, free fatty acids and cholesterol [2]. Substances applied onto the skin surface, thereby, can pass this complex structure by different routes. Although the tortuous pathway between the corneocytes is likely to be the main route through the *stratum corneum*, it can be bypassed by orifices and glands, both of which can account for a large part of the body surface [1]. However, transdermal drug delivery is severely limited to a small percentage of drugs due to physicochemical drug characteristics and barrier properties of the skin. Therefore, considerable effort has been put into the development of sophisticated new transdermal drug delivery systems to overcome the skin barrier. Besides chemical permeation enhancers [3] and electrical techniques of enhancement like iontophoresis and electroporation [4-6], systems like patches and microneedles have been developed for a convenient and effective transdermal drug delivery [7]. Microneedle technology has been established to perforate the skin barrier without inducing pain or bleeding, as the needles are too short to stimulate the nerves and to damage blood vessels in the dermis [8,9]. The needles are made of silicon, glass, metal, polymers or sugar with sizes ranging from sub-micron to millimetres to form microscopic holes that allow enhanced drug delivery [10]. Unlike skin abrasion the microneedle application represents a safe, efficient and controllable alternative for increasing transdermal drug delivery [11].

Over the past few years, four different designs such as “poke and patch” [12], “coat and poke”, “poke and release” [13,14], and “poke and flow” [15] have been fabricated which have already been established for macromolecules like insulin or vaccines [9,12].

The easiest approach of using microneedles is to employ solid microneedles to form a pore in the skin, through which compounds can pass out of the topical formulation [16]. Therefore, two different techniques are disposable: Firstly, solid microneedle arrays are pressed onto the skin or scraped on the skin and secondly, rollers with attached microscopic needles are rolled over the skin. The pores produced by either method are alike, whereby the rollers are easier to use [17]. *In vitro* examinations with solid microneedles have increased skin permeability for substances ranging from nanomaterials to proteins [12,18] concurrent with an increase in the TEWL [10,19].

In the present study, two commercially available microneedle rollers with different needle lengths (200 μm and 300 μm) were utilised with the aim to determine the efficiency of skin perforation and to describe their influence on the permeation of several topically applied non-steroidal anti-inflammatory drugs with different physicochemical drug characteristics in an *in vitro* setup. Moreover, histological staining was performed to characterise the degree of skin perforation after microneedle pretreatment.

## Methods

### Chemicals

All reagents used in the present study were of the highest purity available. Diclofenac (molecular weight (MW): 296 g/mol, logK_o/w_: 0.7, melting point (MP): 284°C), ketoprofen (MW: 254 g/mol, logK_o/w_: 1.8, MP: 94°C), ibuprofen (MW: 206 g/mol, logK_o/w_: 3.97, MP: 76°C), and paracetamol (acetaminophen; MW: 151 g/mol, logK_o/w_: 0.46, MP: 170°C) [20] were obtained from Sigma-Aldrich (Steinheim, Germany). Methanol was purchased from Applichem GmbH (Darmstadt, Germany). All other reagents were obtained from Merck (Darmstadt, Germany).

### Animal skin

The skin was obtained from bovine udders, all of which were harvested from Holstein Friesian cows which died at a slaughterhouse for food production, and the cleaned skin samples were stored at - 20°C until use. After thawing at room temperature split skin samples with a thickness of 600 μm ± 50 μm were produced using an electrical microtome (Zimmer, Eschbach, Germany), whereby damaged skin samples were excluded from the study [21].

### Skin perforation by microneedles

**Figure 1:**
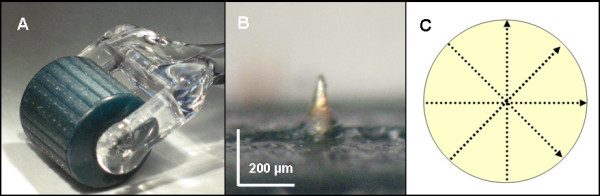
**Microneedle roller.** Representative images of the microneedle roller **(A)** and a 200 μm microneedle **(B)**; **C** shows the microneedle roller application procedure.

Two different microneedle rollers were used (200 μm needle length and 300 μm needle length), both of which possessed of 192 titanium needles (Medik8, London, United Kingdom, Figure 1) in a cylindrical arrangement. Prior to the experiment split skin samples with appropriate size of 2 x 2 cm were incubated in phosphate buffered saline (PBS) for 30 minutes. They were placed on a styropor panel and fixed with needles beyond the subsequent diffusion area of the skin samples before the microneedle rollers were rolled in four axes radial over the skin surface (Figure 1 C).

### Light microscopy

Visualisation of the produced pores was performed by light microscopy. The skin samples were treated with the microneedles as mentioned above and were incubated with saturated methylene blue solution in PBS for 120 minutes. The skin samples were forthwith examined under the light microscope to count the amount of needles pores within the diffusion area. Afterwards, they were frozen and cut in 10 μm thick sections with a cryostat.

### *In-vitro* permeation

The diffusion experiments were performed in Franz-type diffusion cells obtained from PermeGear (Riegelsville, PA, USA) with a receptor chamber of approximately 12 ml and a diffusion area of approximately 1.77 cm². Sonicated PBS was used as receptor fluid. One ml of the following 80% saturated solution in PBS was applied onto the skin samples immediately (within 5 minutes) after pore production: diclofenac 2.6 mg/ml, ibuprofen 23.4 mg/ml, ketoprofen 2.4 mg/ml, paracetamol 17.5 mg/ml. The donor chambers were covered with parafilm® (American Can Company, Baltimore, USA) and were checked for precipitation of the compounds during the whole experiment. Aliquots were taken from the receptor fluid and replaced by the same amount of fresh PBS at 0, 0.5, 1, 2, 4, and 6 hours. Each treatment (untreated control, 200 μm microneedle, and 300 μm microneedle) was performed in duplicate per animal (n = 6).

### Analysis

The receptor fluid samples (100 μl) were analysed by high-performance liquid-chromatography, the methodology of which has derived from recent studies [22]. The following components were obtained from Beckman (Fullerton, CA, USA): autosampler 507, pump 126, and UV–VIS detector 168. The separation took place on a reversed phase column (LiChroCART 125–4, LiChrospher 100 RP-18e, 5 μm (Merck, Darmstadt, Germany)), which was maintained at 40°C. The mobile phase consisted of 80% methanol and 20% McIlvaine citrate buffer (pH 2.2) for both diclofenac and ibuprofen, of 60% methanol and 40% McIlvaine citrate buffer for ketoprofen, and of 15% methanol and 85% McIlvaine citrate buffer for paracetamol. The detection was performed at 282 nm (diclofenac), 238 nm (ibuprofen), 260 nm (ketoprofen) and 245 nm (paracetamol), respectively.

### Data analysis

The results of the diffusion experiment are expressed as mean and standard error. The linear part of the gradient of the permeation curve (time vs. concentration in the receptor fluid) represents the maximum flux J_max_ (μg/cm²/h) and is employed to calculate the apparent permeability coefficient P_app_ (cm/s) according to Niedorf et al. 2008 [23]. Differences between control samples and pretreated skin samples were evaluated by Friedman test followed by Dunn´s multiple comparison test (GraphPad Prism 4.01 (GraphPad Software Inc., San Diego, USA). A 0.05 significance level was adopted.

## Results

**Figure 2:**
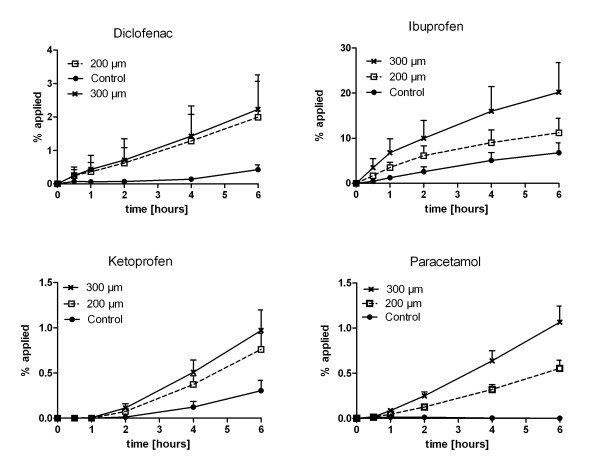
**Permeation profile.** Permeation of diclofenac, ibuprofen, ketoprofen and paracetamol through bovine udder skin samples following pretreatment with 200 μm and 300 μm microneedles in comparison to untreated control skin (n = 5–6); mean + SEM.

The microneedle application results in an enhanced permeation of all applied compounds compared to untreated skin (Figure 2). In skin samples pretreated with the 300 μm microneedles a significant higher permeation was found than in the untreated skin samples, by which the maximum flux (J_max_) and the P_app_-value are up to 3-fold (ketoprofen, ibuprofen) to 7-fold (diclofenac) and 8-fold (paracetamol) higher in the microneedle treated skin samples (Table 1) and hence result in higher recoveries after microneedle pretreatment.

**Table 1:** Permeation parameters

	**Substance**											
	**Diclofenac**			**Ibuprofen**			**Ketoprofen**			**Paracetamol**		
**Parameter**	**Control**	**200 μm**	**300 μm**	**Control**	**200 μm**	**300 μm**	**Control**	**200 μm**	**300 μm**	**Control**	**200 μm**	**300 μm**
J_max_ (μg/cm²/h)	1.72	8.75	11.55	167.78	368.69	431.52	1.03	2.36	2.99	2.58	9.97	19.42
10^-6^ P_app_ (cm/s)	0.18	0.93	1.24^*^	1.92	4.21	5.27^*^	0.12	0.28	0.35^*^	0.04	0.16	0.31^*^
Recovery (%)	0.49	3.17	3.54	6.76	11.15	17.75	0.30	0.76	0.96	0.14	0.55	1.06

Mean permeation parameters following substance application to bovine skin; * = p<0.05 (microneedle versus control), n = 5–6.

The correlation of physicochemical drug characteristics with the enhancement of the permeation reveals that substances with low lipophilicity (R² = 0.73) and high melting points (R² = 0.76) benefit from microneedle application, while there is no correlation of the microneedle pretreatment to the molecular weight (R² = 0.01).

**Figure 3:**
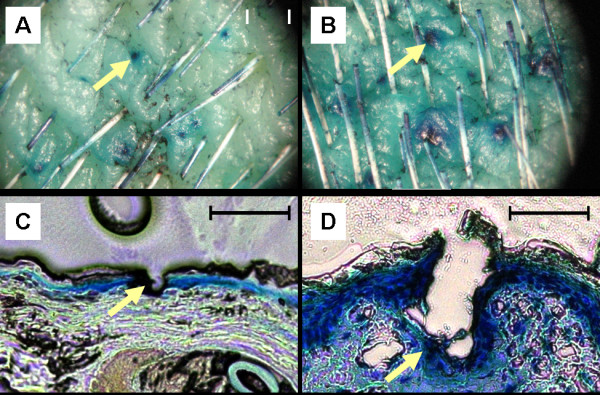
**Microneedle treatment.** Light microscopic images of bovine skin treated with microneedles of 200 μm **(A)** and 300 μm **(B)** needle lengths after topical administration of methylene blue solution for 6 hours; the arrows show the punctured areas with methylene blue penetration into deeper skin layers. C and D show histological images of perforated skin samples **(C:** 200 μm, **D:** 300 μm) after administration of methylene blue on the microneedle pretreated skin samples; the arrows show the punctured areas with methylene blue penetration into deeper skin layers. The bars represent 500 μm.

Although no visible pores are detectable by light microscopy directly after puncturing the skin, the methylene blue application reveals the existence of barrier damage after microneedle treatment (Figure 3 A and B), which is also detectable in histological sections (Figure 3 C and D). The density of the microscopic holes was approximately 48 pores/cm².

## Discussion

In the present study, two commercially available microneedle rollers with different needle lengths have been utilised to overcome the natural skin barrier. A staining method was employed to determine the ability of the microneedles to invade into the skin, and diffusion experiments with several non-steroidal anti-inflammatory drugs were performed to investigate the ability of microneedles to enhance transdermal drug delivery of non-steroidal anti-inflammatory drugs.

At first, the capability of the microneedle rollers to disrupt the skin barrier could be confirmed by the blue staining under the light microscope. Methylene blue is a dye with a molecular mass of 320 g/mol with a high affinity to proteins. The latter characteristic results in the fact that after application of methylene blue solution onto physiological intact skin no dye can be found in deeper skin layers. Thus, the methylene blue staining made the non visible pores after microneedle pretreatment detectable.

As the needle assembly, the geometry and the velocity insertion of the microneedles treatment [24] severely influence the penetration depth and the pore size, a direct comparison between various types of microneedles should be made with caution. However, in accordance with former studies which demonstrated that 150 μm long needles do not form measurable wholes in the skin [10], the 200 μm and 300 μm needles show similarly manners. As a result of an *in vitro-*study*,* information about pain or bleeding could not be determined. Since no alterations in the deeper skin layers have been observed (Figure 3 C and D), it is likely that the needles used in the present study can not cause any pain or bleeding, and recent studies in humans have demonstrated that microneedles were applied to human skin in a painless manner [8,25].

Moreover, the ability of microneedles to enhance skin permeability of non-steroidal anti-inflammatory drugs was verified by *in vitro-*diffusion experiments with bovine split skin. The application of two types of microneedles resulted in altered permeation rates for both needle lengths, yet only the 300 μm-microneedle roller led to a statistical significant higher permeation rate for all test compounds. This may be due to the considerable barrier disruption produced by the 300 μm needles and may be adjusted by increasing amounts of pores of the 200 μm needles. However, higher amounts of pores intensify skin permeability only for a certain extent [26]. For microbiological risk assessment, an *in vitro*-study has been performed after microneedle administration by Donnelly et al. 2009 [27]. It has been shown that microneedle induced holes in the *stratum corneum* result in significant less microbial penetration than hypodermic needles and no microorganisms crossed the viable epidermis after microneedle pretreatment. Thus, it is likely that microneedle application in an appropriate manner will not result in either local or systemic infections in immune-competent individuals as far as the microneedles are manufactured under aseptic or sterile conditions [27].

Recent *in vitro*-examinations about permeation enhancement of topically applied substances in microneedle treated skin revealed a permeation enhancement up to 2 times for the hydrophilic acetylsalicylic acid [28], whereas the previous study demonstrated permeation enhancements up to 3–8 times depending on the substance lipophilicity. But it has to be taken into consideration that the manner of application of the needles complicates a direct comparison between different examinations as well as the skin type used (full thickness skin vs. split skin) [10].

In order to obtain information about the influence of physicochemical drug characteristics on drug enhancement by microneedles, non-steroidal anti-inflammatory drugs with different molecular weights, lipophilicities and melting points were chosen. Despite the application of 80% saturated solutions for each compound, different levels of permeation enhancement were obtained. In contrast to a comparative study with different particle sizes which demonstrated that small sizes were more effective in drug delivering into the horny layer [10] the present study did not reveal a correlation between permeation enhancement and molecular weight. However, a higher permeation enhancement was observed for more hydrophilic compounds like paracetamol and diclofenac compared to the lipophilic drugs ibuprofen and ketoprofen. This may be due to the effect that hydrophilic substances, that bypass the lipophilic *stratum corneum* e.g. by a microscopic pore, partition faster into the hydrophilic skin layers compared to lipophilic compounds [7,10,29-34]. Once a hydrophilic drug has bypassed the lipophilic *stratum corneum* a fast permeation into the receptor fluid can be assumed, since the dermis does not represent a distinct barrier for hydrophilic compounds [35].

Another important physicochemical drug characteristic in transdermal drug delivery is the melting point of the applied compound [36,37]. Substances with low melting points exhibit a high solubility in epidermal lipids, which in turn provides a higher thermodynamic activity for percutaneous permeation. Hence, it is not surprising that the present study reveals a higher permeation enhancement for substances with high melting points (diclofenac and paracetamol), both of which can bypass the *stratum corneum* lipids through the pores produced by the microneedles.

Previous *in vivo-*investigations performed by Bal et al. 2008 [38] showed that under non occlusive conditions the pores remained open for a few hours, which can be enhanced up to 72 hours by performance of occlusive conditions [39]. Since the present study was conducted under occlusive conditions, it is likely that the pores have been open for the entire experiment. Furthermore, barrier disruption can result in a fast substance influx into the deeper skin layer with depot formation. This depot can release the substance into the blood or lymphatic system *in vivo*.

## Conclusion

The present study demonstrates the ability of 200 μm and 300 μm long microneedles to interrupt the main skin barrier and to enhance transdermal drug delivery of topically applied non-steroidal anti-inflammatory drugs especially with a hydrophilic nature and high melting points by orders of magnitude. This transdermal delivery approach is easy to employ, minimally invasive and represents an appealing method with great potential for other applications.

## Competing interests

The authors declare that they have no competing interests.

## Acknowledgements

The authors acknowledge the help given by Bettina Blume with respect to the acquisition of the bovine udder skin and Theiss Wystemp and Victoria Garder for technical help.

## Authors’ contributions

JS designed the study, conducted the histological examinations, contributed to the analysis, interpreted results and drafted the manuscript. MW participated in the diffusion experiments. MK participated in the study design development. All authors read and approved the final manuscript.

## Pre-publication history

The pre-publication history for this paper can be accessed here:

http://www.biomedcentral.com/2050-6511/13/5/prepub
